# Inflammation-associated enterotypes, host genotype, cage and inter-individual effects drive gut microbiota variation in common laboratory mice

**DOI:** 10.1186/gb-2013-14-1-r4

**Published:** 2013-01-24

**Authors:** Falk Hildebrand, Thi Loan Anh Nguyen, Brigitta Brinkman, Roberto Garcia Yunta, Benedicte Cauwe, Peter Vandenabeele, Adrian Liston, Jeroen Raes

**Affiliations:** 1 Department of Structural Biology, VIB, Pleinlaan 2, 1050 Brussels, Belgium; 2 Department of Bioscience Engineering, Vrije Universiteit Brussel, Pleinlaan 2, 1050 Brussels, Belgium; 3 Autoimmune Genetics Laboratory, VIB, Herestraat 49, 3000 Leuven, Belgium; 4 Katholieke Universiteit Leuven, Herestraat 49, 3000 Leuven, Belgium; 5 Department for Molecular Biomedical Research, VIB, Technologiepark Zwijnaarde 927, 9052 Ghent, Belgium; 6 Department for Molecular Biomedical Research, GhentUniversity, Technologiepark Zwijnaarde 927, 9052 Ghent, Belgium

## Abstract

**Background:**

Murine models are a crucial component of gut microbiome research. Unfortunately, a multitude of genetic backgrounds and experimental setups, together with inter-individual variation, complicates cross-study comparisons and a global understanding of the mouse microbiota landscape. Here, we investigate the variability of the healthy mouse microbiota of five common lab mouse strains using 16S rDNA pyrosequencing.

**Results:**

We find initial evidence for richness-driven, strain-independent murine enterotypes that show a striking resemblance to those in human, and which associate with calprotectin levels, a marker for intestinal inflammation. After enterotype stratification, we find that genetic, caging and inter-individual variation contribute on average 19%, 31.7% and 45.5%, respectively, to the variance in the murine gut microbiota composition. Genetic distance correlates positively to microbiota distance, so that genetically similar strains have more similar microbiota than genetically distant ones. Specific mouse strains are enriched for specific operational taxonomic units and taxonomic groups, while the 'cage effect' can occur across mouse strain boundaries and is mainly driven by *Helicobacter *infections.

**Conclusions:**

The detection of enterotypes suggests a common ecological cause, possibly low-grade inflammation that might drive differences among gut microbiota composition in mammals. Furthermore, the observed environmental and genetic effects have important consequences for experimental design in mouse microbiome research.

## Background

An accumulating body of evidence supports the central role of the intestinal microbiota in maintaining its host's health. Dysbiosis of the gut microbiota is linked to many chronic disorders [1], such as inflammatory bowel disease [2-4], obesity [5-7], rheumatoid arthritis [8], autoimmune encephalomyelitis [9,10], type 1 [11,12] and type 2 diabetes [13], and allergic diseases [14].

The gut flora composition is known to vary among healthy individuals [15-18], along the intestinal tract [19-21], and over time [22,23]. Although the factors influencing the species composition and functionality of the healthy gut flora are still being revealed, food [24-26], drug uptake [14,27], inoculation at birth [28,29], host genetics [6] and as yet unknown environmental factors all seem to play a role [30]. Concomitantly, the intestinal microbiota plays an important role in shaping the host's immune system [8,31,32] and physiology [5,8,33].

Due to limitations of human research, the details behind many of these processes are still unknown. Therefore, murine models have become crucial in gut microbiota research for gaining mechanistic insights into gut flora establishment and upkeep. Such models can be used to investigate the effects of food and drug uptake or the interplay between host and microbiota, demonstrating causality in disease and therefore the relevance of these model systems [34-37]. Knock-out and transgenic models have shown that host genes can influence the microbiota composition [38-41], have given insights into signaling cascades that mediate microbiome-host interactions [31,32,42,43] and enabled the study of the interplay between host physiology and microbiota composition [44-46].

However, various confounding factors can hamper the interpretation and comparison of community shifts in rodent model research. Among these are cage effects [47,48], inter-individual variation [22,49], genetic background [50-52] and maternal effects [50,52,53]. Here, we present data regarding the relative contribution of cage effects, genetic background and inter-individual variation to the murine microbiota in laboratory mice in a mixed co-housing design. Using 16S rDNA pyrosequencing-based profiling, we determined the baseline species composition of five different strains, investigated enterotype stratification and quantified the relative contribution of genetic and environmental effects to the overall variation of the murine gut microbiota. Finally, we discuss the consequences of our findings for the experimental design of microbiota studies in murine disease models.

## Results

### Experimental set up

To study inter-individual variation and the influence of genetic and environmental components on gut microbiota composition, we investigated the flora of five mouse strains commonly used in biomedical research: four inbred (Balbc, B6, FVB and non-obese diabetic (NOD)) and one out-bred strain (Swiss). Five female mice (one from each strain) were co-housed together for 3 weeks after weaning, and this setup was replicated ten times. The 3-week period of co-housing aimed at minimizing the effects of parent cages from which the mice came from. Furthermore, we investigated the impact of sex by a weekly transfer of used bedding from each cage of female mice to a corresponding cage housing a male B6 (one per cage; ten replicates) to replicate the environmental conditions without direct physical contact. After the co-housing period, mice were sacrificed and DNA was extracted from the cecal content. The V3-V5 variable region of 16Sr RNA genes was amplified by PCR [54-56] (see Materials and methods) and the amplicons were sequenced using 454 pyrosequencing.

### Bimodal distribution in mouse microbiome composition: evidence for two enterotypes with significantly different species richness across investigated mouse strains

**Figure 1:**
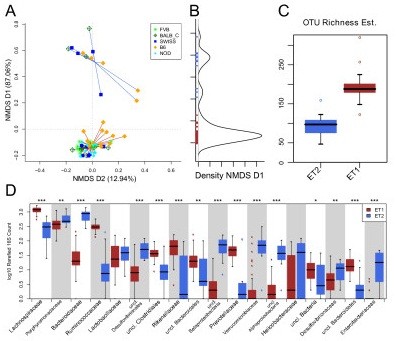
**Enterotype clusters detected in the data**. **(a) **Nonmetric multidimensional scaling (NMDS) at the genus level shows two clusters in the dataset. **(b) **The density of the first NMDS axis that explains most of the variation (92.5%) and shows a bimodal distribution with only few intermediate samples. **(c) **The operational taxonomic unit (OTU) richness estimate (chao1) between these two clusters differs substantially and **(d) **the two clusters are dominated by different taxa, with enterotype 2 being dominated by Bacteroidetes and Enterobacteriaceae and enterotype 1 being driven by Runinococcus and Lachnospiraceae. Significances are shown by asterisks: *q < 0.1 and *P *< 0.05; ** q < 0.05; ***q < 0.01.

For the majority of the samples, Firmicutes (58.64 ± 23.53%) and Bacteroidetes (35.21 ± 19.0%) were the two main phyla that dominated the gut community (Additional file 1). Other phyla such as Verrucomicobia, Proteobacteria and Tenericutes together comprised, on average, less than 5% of total community composition, in line with previous reports [5,57]. Our initial sample clustering showed a strong sample separation into two separate clusters (Figure 1a; Additional file 2), with multiple genotypes occurring in each cluster. Both male and female mice were found in each cluster and the male/female ratio was not significantly different between the smaller (0.2) and the larger cluster (0.16) (*P *= 1, Fisher's exact test). No significant association between clusters and cages was found (*P *= 0.701, permuted Fisher's exact test; Additional file 3). Not all mouse strains were represented in the smaller cluster: NOD and FVB did not have any individuals in the second cluster. Given our sample size, however, it could not be determined if this absence was a true biological trend or was due to random chance.

Given the similarity to the enterotypes found in the human population [16], we tested whether the two clusters fitted the criteria used in the original study. The optimal cluster number was found to be two by both the Calinski-Harabasz (CH) index as well as silhouette score, independent of distance metric used (Additional file 4); the silhouette score (ranging from 0.6 to 0.825 at all levels except the operational taxonomic unit (OTU)) indicates strong evidence for independent clusters [58] and the density of individual mice along the first nonmetric multidimensional scaling (NMDS) axis shows a bimodal distribution (Figure 1b), with possibly 3 of 60 samples being intermediate. This was further confirmed using two additional optimal cluster score algorithms: Baker and Hubert Gamma and Davies-Bouldin's index (Additional file 5). Additionally, we tested several different clustering algorithms, including k-means clustering, as well as average, single, ward and complete hierarchical clustering, all pointing to two optimal clusters (Additional file 5). To further assess the robustness of these clusters, we randomly (i) jackknifed the samples and (ii) resampled the taxonomic assignments 500 times, and could recover in 100% of cases two clusters using the Silhouette index under all tested conditions. The only exception to this was that the CH index showed a weak possibility for three clusters using the Bray-Curtis distance (weighted Unifrac and Jensen-Shannon distance gave two as the optimal number of clusters (CH index) in > 98% of cases; Additional file 6). Taxonomic resampling showed that the three intermediate points (Figure 1b) can switch their cluster identity, possibly also explaining the (weak) support for three clusters in some settings.

One of the two clusters showed a significantly lower richness and diversity compared to samples in the other cluster (Figure 1c), reminiscent of recent reports of diversity differences between enterotype-like subpopulations in a human cohort [59]. In addition, we found that in the smaller low-richness cluster, the proportion of Firmicutes was largely reduced (from an average of 68.9% to 17.5%) while Bacteroidetes (27.4% to 65.6%) and Proteobacteria (1.6% to 12.5%) were highly increased. All these changes were strongly significant (Additional file 7). The most affected families from the decrease in Firmicutes were Lachnospiraceae and Ruminococcaceae (Figure 1d), which contributed 43.8% and 11.4%, respectively, of the total composition. By contrast, in the low richness samples, multiple families of Proteobacteria were significantly increased in their abundance (*P *< 0.05 and q < 0.1), including Enterobacteriaceae. The other two families also found to be strongly enriched were Porphyromonadaceae and Bacteroidaceae, both of which are generally dominant members of the murine gut microbiota (on average 20.3% and 8.4% of the overall community, respectively). These compositional and community structure properties of the two detected clusters, enterotype 1 (ET1) and enterotype 2 (ET2), are strikingly similar to those of the *Ruminococcus *and *Bacteroides *enterotype found in human populations, respectively [16].

### Enterotypes associate with low-grade inflammation

**Figure 2:**
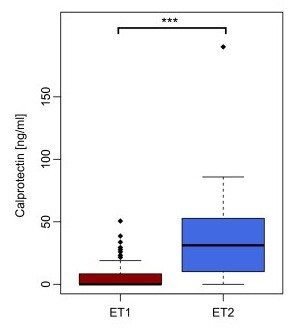
**Calprotectin concentration (ng/ml) in enterotype 1 (ET1) and enterotype 2 (ET2)**. An elevated concentration of calprotectin was found in Bacteroidetes dominant enterotype (ET2) (*P *= 4.9 × 10^-5^, Wilcox rank sum test).

To further investigate the biological reasons behind this clustering, we assessed the level of intestinal inflammation using calprotectin levels in their cecal content [60,61]. Mice in the low richness group had significantly increased fecal calprotectin levels (*P *= 4.9 × 10^-5^, Wilcox rank sum test) compared to the high richness samples (Figure 2). Calprotectin levels were significantly negatively correlated to Lachnospiraceae, Rikenellaceae, Ruminococcaceae as well as Prevotellaceae, while the positive correlation to Bacteroidaceae, Verrucomicrobiaceae, Enterobacteriaceae and Burkholderiales was significant (Additional file 8). The low richness mice showed no obvious signs of inflammation or disease, suggesting a low grade inflammatory condition.

### After enterotype stratification, genetic, cage and inter-individual variation effects contribute on, average, 19%, 31.7% and 45.5% to the variance in the murine gut microbiota composition, respectively

**Figure 3:**
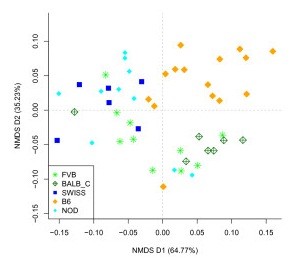
**NMDS plot of enteroype 1 stratified sample set at the phylum level**. Samples are colored by mouse genotypes and the percent of variation explained by each axis is indicated in parentheses.

To further investigate the effect of the host's genetic and environmental properties on microbiota composition, we stratified our population according to the two enterotypes described above and focused on the largest group (ET1). The overall community composition was significantly associated with both genetic and cage effects, as tested by PERMANOVA (*P *= 2 × 10^-7 ^and *P *= 2 × 10^-7^, respectively, on the OTU level; Table 1). A NMDS ordination was used to visualize these effects. At the phylum level the mice formed approximate clustering according to their genotype, as shown in Figure 3. Visually inspecting ordinations on different taxonomic levels revealed that the strength of genotype-associated clustering decreased with more fine-grained taxonomic levels, while the significance of the cage effect increased concomitantly (Table 1; Additional file 9). These trends were further confirmed using distance-based redundancy analysis (dbRDA, Additional file 10). Comparing the gut microbiota of male and female B6 mice revealed that there was no significant sex effect observed in our bedding-exchange design (*P *= 0.12; Table 1).

**Table 1:** PERMANOVA test for significance of factors contributing to overall differences in microbiota composition

	Phylum	Class	Family	Genus	OTU
Genotype	2.00E-07	2.00E-07	2.00E-07	2.00E-07	2.00E-07
Cage	0.0238	5.08E-05	7.26E-05	6.20E-06	2.00E-07
Sex	0.44	0.2972	0.3003	0.3926	0.2456
Sex_block	0.374	0.09805	0.1091	0.2072	0.05531

Genotype and cage were significantly associated with differences in microbiota composition whereas sex did not have an effect. Note that only B6 mice were used to test for sex effects as we only had females and males from B6. We repeated the PERMANOVA test for the complete dataset, using genotype in a blocked design to test for sex effects (sex_block). We used 5 × 10^7 ^permutations to calculate the significances.

Next, we determined the percentage of variance that can be explained by both genetic background and cage effects at different taxonomic levels using variation partitioning (see Materials and methods; Table 2). Also here, a decreasing fraction of the variance could be explained by genotype when going from phylum (26.55%) to OTU (15.65%) level. Conversely, cage effects showed an opposite trend, with higher variation explained at low levels such as OTU, genus, family and class (above 31%) and the smallest effect at the phylum level (22.6%). Overall, these results show that both host genetic and cage effects have a strong influence on microbiota composition, explaining, on average, 19% (genotype) and 31.34% (cage) of the variation. The shared variation explained by genotype and cage effects was small (from 1.35% to 7% explained variance) compared to the influence of genotype or cage effects alone, suggesting independent effects on the microbiota composition. Stochastic and inter-individual effects still contributed the largest part to the variation that drives differences between the murine microbiome, explaining from 42.1% to 51.13% of the variation when stratifying for enterotypes. If the variation explained between cage, genotype as well as enterotype is calculated, enterotype explains the largest part of the variation (25 to 27%) on most taxonomic levels (Additional file 11).

**Table 2:** The percentage of variation explained by factors influencing microbiota composition

	Phylum	Class	Family	Genus	OTU
Genotype	26.55	18.62	18	16.64	15.65
Genotype and cage	7.01	4.61	3.73	3	1.35
Cage	22.6	34.68	32.39	35.17	31.87
Other	43.84	42.1	45.88	45.18	51.13

'Genotype and cage' denotes variation explained by both factors. 'Other' implies all variations that could not be unaccounted for. These data were stratified for the larger enterotype. Genotype and cage effects were significant at all phylogenetic levels.

In addition, we tested if the within-strain variability differed between strains. However, there was no significant difference on any taxonomic level (Additional file 12). Thus, it appears that within-strain variation is comparable irrespective of the genotype.

### Positive association between genetic distance and microbiota profile

**Figure 4:**
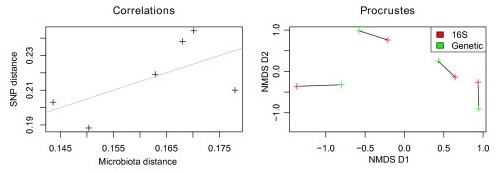
**The genetic distance between mouse strains is significantly correlated to phylum level microbiota distances**. A Procrustes superimposition of the NMDS of both data types shows a clear association between mouse genotypes and microbiota composition. The *P*-value is calculated separately with a Mantel test.

We further investigated genotype-microbiota association by correlating genetic distance to microbiome composition of investigated mouse strains. A recent genetic analysis of a broad range of laboratory mouse strains [62] included four out of five strains used in this study (B6, Balbc, FVB and NOD). Based on these data, we found a significant positive association between genetic distance and the average microbiota distance at the phylum level (rho = 0.606, *P *= 0.037; Figure 4) and genus level (rho = 0.65, *P *= 0.042), which confirms the presence of a genetic effect on gut microbiota composition.

The pattern of different levels of similarity between the individual strains was further investigated using a PERMANOVA *post hoc *test. Generally speaking, most strains were significantly different at all phylogenetic levels, except for Swiss and NOD (all levels), FVB and Balbc (only significant at the OTU level) and Swiss and FVB (only significant at the phylum and OTU levels; Additional file 13). This result reflects the phylogenetic relationship of mice found in [62], in which FVB and Balbc were very similar to each other whereas other groups were more distant (for example, FVB versus B6).

When investigating alpha diversity patterns, we observed significant OTU richness differences between the genotypes (*P *= 0.0263), but no significant differences between cages could be detected (*P *= 0.269). FVB showed the lowest OTU richness while NOD had the highest richness of all strains. In line with this, Chao1 richness estimates were significantly different between genotypes (*P *= 0.011), but not between cages (Additional file 14). In a *post hoc *test the differences between FVB and Swiss, NOD and Balbc were significant after multiple testing (*P *< 0.01, q < 0.04). However, estimates of diversity (which also takes into account community structure) showed no significant differences between either genotypes or cages.

### Bacterial genera driving differences between genotypes and cages: identification of *Helicobacter *as an important driver of cage effects

**Figure 5:**
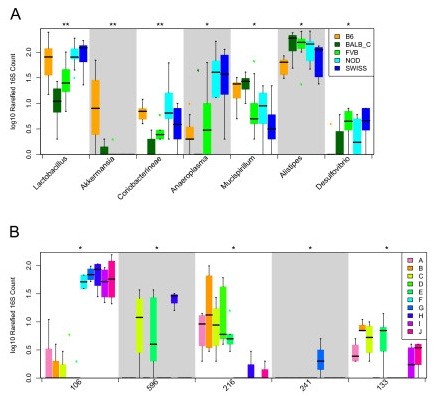
**Taxa differences between genotypes and cages**. **(a) **Several genera are significantly different in abundance between genotypes, with a cutoff of *P *< 0.05 and q < 0.1. **(b) **The significantly different OTUs between cages. On the y-axis log10 scaled rarefied 16S reads per sample are shown. OTU identifiers refer to the following taxonomic assignments: 106 = *Helicobacter*; 596, 216, 133 = Porphyromonadaceae; 241 = *Sphingomonas*.

To further understand the genetic and environmental effects on microbiota composition, we studied the phylogenetic profiles of the different strains and cages in more detail. Figure 5 shows the groups that were significantly different between genotypes (*P *< 0.05, q < 0.1; Additional file 15). *Akkermansia *(0.56% of total composition versus 0.025 on average in other strains), *Lactobacillus *(2.6% versus 1.67%) and *Mucispirillum *(0.59% versus 0.34%) were enriched in B6 mice, whereas only *Mucispirillum *(0.65% versus 0.33%) was overrepresented in Balbc mice. *Desulfovibrio *was significantly increased in FVB and Swiss mice (0.10% and 0.11% versus 0.03%). On the other hand, Swiss mice showed higher levels of *Anaeroplasma *(1.30%), *Lactobacillus *(2.84%) and *Desulfovibrio *(0.11%) but showed a significant reduction in *Mucispirillum *(0.14%) compared to B6, Balbc and FVB (average of 0.18%, 1.25%, 0.5% and 0.51%, respectively; Additional file 16). One notion from this comparison was that NOD and Swiss mice were similar throughout these comparisons, and this corresponds to the results of the multivariate analysis showing no strong differences between NOD and Swiss mice. Interestingly, *Akkermansia*, a well-known mucin degrader [63], could not be detected in NOD and Swiss mice but was very abundant in B6 (Figure 5a). Finally, although NOD mice are known to develop spontaneous diabetes and were expected to have different microbiota composition, we did not see very striking NOD-specific microbiota shifts in this study.

Additional file 17 lists taxonomic groups that were significantly different among cages. Several Bacteroidetes subgroups (*Sphingomonas*, unclassified Parabacteroides, unclassified Prevotellaceace, unclassified Porphyromonadaceae and *Sporobacter*) as well as an unclassified Proteobacteria group seemed to be linked to the cage effect but did not withstand multiple testing correction (*P *< 0.05, q > 0.1). The only significantly different genus between cages was *Helicobacter *(Kruskal-Wallis test *P *= 0.00021 and q = 0.0097), a well-known and fast spreading species in mouse facilities [64] that was overrepresented in five out of ten cages studied (Figure 5b). *Helicobacter *levels varied on average by 0.011% to 2.15% among cages. The abundance of *Helicobacter *was not significantly different between genotypes (*P *= 0.56) nor was it different among the two enterotypes described above (*P *= 0.053). At the OTU level, five OTUs were significantly different between cages; three belonged to *Porphyromonadaceae*; *Helicobacter *and *Sphingomonas *had one representative each (Figure 5b).

To determine the contribution of *Helicobacter *to the total cage effect, we artificially removed all *Helicobacter *OTUs from the data. From this we could derive that *Helicobacter *contributed approximately 6% to the total variation between cages at the genus level. However, at higher taxonomic levels (family and class) the percentage of variance solely explained by *Helicobacter *increased to 10% and 13%, respectively. PERMANOVA results confirmed that the cage effect was only significant at the genus and OTU levels (Additional file 18) if *Helicobacter *was removed from the data. The removal of *Helicobacter *OTUs had no effect on the significance of the genotype effect.

Performing the univariate tests on 60 samples in a blocked Kruskal-Wallis test using enterotype as a confounder instead of pre-stratifying gave largely the same results (Additional file 19).

## Discussion

In this study we compare the healthy mouse microbiome of different common laboratory strains. We identified two distinct enterotype-like subpopulations in our study group, separated by richness and independent of strain and cage. Stratifying for these two populations, we show the impact of genetic versus environmental factors on the murine gut microbiota.

The strongest signal separating our dataset is the presence of two enterotype clusters, different in species composition and diversity, which were strongly supported using multiple metrics and evaluation criteria. The phylogenetic composition is highly similar to two of the recently described human enterotypes: the low richness cluster is dominated by Bacteroidetes, while the high richness cluster is dominated by Ruminococcaceae and several other genera, which suggests that the two clusters found here might overlap with the first and third human enterotype and may possibly be influenced by the same ecological drivers. Furthermore, these results agree with the observed difference in diversity between Firmicutes- or Bacteroides-dominated subgroups found in a human cohort [59]. All these observations suggest that enteroype-like community structures exist in laboratory mice and that their ecology might be similar to that of the human microbiota, despite the known gut microbial compositional differences between human and mouse, suggesting that enterotypes are possibly a universal feature across mammals.

As low species richness was observed in obese people [6] and inflammatory bowel disease patients [65,66], in which it was associated with inflammation signs in the host, we suspected that the low richness observed here might be linked to (low-grade) inflammation as other confounding factors, such as diet [26], were accounted for in this setup. Another indication came from a very significant increase in Enterobactericeae in the low richness cluster, a group that has been associated with induction of low grade inflammation through lipopolysaccharide [67]. Indeed, calprotectin levels were increased in the low richness enterotype samples, confirming our hypothesis. Likewise, a recent study employing a colitis susceptible model showed that inflamed mice had a lowered gut microbiota richness as well as increased Enterobacteriaceae abundance [68]. The observation that low-grade inflammation can occur in young, healthy specific-pathogen-free (SPF) mice provides a first hypothesis of the occurrence of the second enterotype. Whether inflammation, low richness or the specific bacterial composition of the low richness enterotype (including inflammation-inducing genera) is causal to the other is unclear from our data - also, a combination of cause and consequence (for example, inflammation contributing to a more inflammatory microbiota) is possible. Further studies using larger quantities of mice for each strain, in conjunction with detailed immunological profiling, possibly with a time-series design, will be needed to fully disentangle the ecology behind the observed groups. Such studies will also be able to determine whether the enterotypes are discrete entities or reflect ecological gradients [69], as the enterotype concept does not exclude gradient behavior [16]. In this regard, the three intermediate samples in Figure 1a that are unstable in cluster identity upon resampling are of particular interest. They could (i) represent a stable state existing between the two main enterotypes determining a third cluster, (ii) stably lie on a less populated ecological gradient between ET1 and 2, or (iii) represent a temporarily unstable state between the two enterotypes (that is, be 'underway' from ET1 to ET2).

The second, *Prevotella*-associated enterotype, as described in human [16,26], was not detected in our data. This absence might be due to the fact that the *Prevotella *enterotype has been the least prevalent enterotype [16] and our sample size might not be big enough to capture it. In addition, the abundance of this genus has been shown to be sensitive to food intake in both humans [26,70,71] as well as in mice [72] - the uniform nutrition within our experimental setup might have hampered the observation of this third type. This said, there is no *a priori *need to observe three enterotypes in mouse and the *Prevotella *type might be human-specific. Future experiments with larger sample size that include diet variation should be able to resolve this issue.

We find that genetic effects influence the composition of gut microbiota in five mouse strains that are commonly used in biomedical research. Although microbiota differences between strains have been observed before [50,51], this is the first study that takes into account the interaction between genetic background and micro-environment as well as other stochastic effects that shape gut microbiome composition. Furthermore, the depth of resolution provided by 16S rDNA pyrosequencing enabled us to quantify its contribution to the overall variation and identify multiple lineages associated with each mouse strain. We found that the genetic effect is strongest at the phylum level (26.55%) and comprises up to 15.65 to 18.62% of the explained variation in the microbiome at lower phylogenetic levels. Thus, it appears that host genetics is influencing the gut metagenome mostly at higher phylogenetic levels. From an evolutionary point of view this strategy is more plausible as broad-spectrum control based on conserved features would be more efficient. Furthermore, these observations are in line with the recent report of higher phylogenetic level control of gut microbiota composition by variable, strain-specific α-defensin expression [73,74]. Likewise, bile acid secretion was shown to affect gut microbiota composition mostly at phylum level [75], and its secretion rate as well as pool size varies between genotypes [76,77]. These observations provide first mechanistic hypotheses why host genetic control would mainly act at higher levels.

Not only is the microbiota composition significantly different between mouse strains, but we find evidence that genetic similarity is correlated to microbiome similarity. This implies that polygenetic markers actively influence gut microbiota composition, and with higher divergence between strains, these as yet unidentified loci are subjected to divergence. However, this distinction was only possible on the phylum and genus levels, as our work was limited by the number of strains available for comparison and a greater number of strains would be required to establish the exact nature of the genetic-microbiota distance relationship.

In addition, we show that the cage effect accounts for a large fraction (up to 30%) of the observed variance in microbiome studies, which has important consequences for experimental design. Our results suggest that the gut microbiota of mice within each cage synchronize to a limited degree and thus influence study outcomes. Indeed, two recent studies [48,78] demonstrated that microbiota-related phenotypes can be transferred between co-housed mice after several weeks of sharing a cage. Here, we observed that this can even happen across different strains, showing the strength of this effect. Gastrointestinal tract synchronization is likely achieved through coprophagy [47]; however, this has not been proven so far. This means that in a typical cross-sectional experimental design, the groups of interest should be kept in a mixed microenvironment, that is, in the same cages, or be separated individually. Otherwise, seeming differences between groups could be solely due to microbiota synchronization within the to-be-compared groups within the same cage. Although a mixed set-up might cause the non-detection of weaker signals because of synchronization between the case and control groups, it does give more weight to signals that are detected against this counteracting force. As reported in this study, the cage effect has the strongest influence on lower taxonomic levels. Thus, studies focusing on microbial differences at the strain level need to take special care to account for within-cage synchronization. In our dataset we identified *Helicobacter *as one of the main drivers of the cage effect, a genus found in other studies to be a sensitive component of the environment [64]. *Helicobacter *is inherently able to overcome the acid gut barrier and thus a steady influx of *Helicobacter *through coprophagy might help this genus to establish in co-caged, unaffected individuals.

Next to an important contribution by (stochastic) individual variation, we show that both genetic and cage (environmental) effects influence the gut microbiota, with the cage effect explaining a slightly bigger fraction of the variance. While the cage effect becomes more important at lower phylogenetic levels, the genetic effect is more important at the higher phylogenetic levels; thus, it appears that the strength of these effects varies in opposite directions along the gradient of taxonomical resolution.

## Conclusions

We show first evidence for the existence of enterotypes in mice as found in humans, suggesting that bacterial gut communities converge into a limited set of stable states, possibly driven by or even contributing to inflammation. Furthermore, our results also show the influence of genetic background and environment on laboratory mouse microbiota composition, stressing the importance of careful experimental design and population stratification before or during analysis. This work underscores the great complexity of host-environment-microbiota interactions, but also brings us one step closer to untangling this fascinating interplay.

## Materials and methods

### Mice

The mouse strains (genotypes) Balbc (BalbCAnNCrl), B6 (C57Bl/6 JCRL), Swiss Webster and FVB (originally from Taconic) were provided by the mouse house of the KU Leuven (KUL). In-bred mice were purchased from vendors and being maintained in the KUL's mouse house by sibling breeding. The NOD mice were originally purchased from the Jackson Lab in 2009 and have been maintained by sibling breeding. Of the five strains used, only Swiss Webster was out-bred whereas the others were in-bred strains. At the beginning of the experiment, mice were age-matched at the age of 4 weeks except for NOD mice, which ranged from 4 weeks old (2 mice), and 6.5 weeks old (3 mice) to 9 weeks old (5 mice). As we did not observe significant differences in microbiota composition between age groups (data not shown), in accordance with previous studies [40], we considered this group as homogeneous and suitable for the study at hand.

Females from each strain were housed together in one cage for 3 weeks. A corresponding male cage containing one B6 male received the bedding from a corresponding female cage every week. Ten replicates from each group were performed. The mice were housed in specific-pathogen-free (SPF) conditions with a 12 hour light/dark cycle. All mice were sacrificed the same day at the age of 8 to 12 weeks. Of the ten NOD mice used, only one developed diabetes at the age of 12 weeks. The experiment followed ethics protocols approved by the University of Leuven Animal Ethics Committee.

### Cecal DNA extraction

Cecal content was collected, resuspended in 1.5 ml Qiagen (Venlo, The Netherlands) stool kit ASL buffer and immediately frozen at -80°C until further analysis. DNA from the samples was extracted using the QIAamp DNA Stool Mini Kit (Qiagen) with adaptations [79].

### PCR amplification of 16S rDNA genes

16S amplification was described previously [41]. Briefly, the V3-V5 region of 16S rDNA genes of the bacteria population were amplified using two primer sets designed for 454 sequencing [56]. The reverse primer of the set contained the 454 adaptor sequence, allowing coupling of the DNA to sequencing beads, a four nucleotide key sequence (TCAG), unique Molecular Identifier (MID) sequences to label each sample (Additional file 20) and the 926 reverse primer sequence (5'CCGTCAATTCMTTTRAGT 3'). The forward primer included the alternative 454 adaptor, a four nucleotide key sequence (TCAG) and the 357 forward primer sequence (5'CCTACGGGAGGCAGCAG 3'). Two 454 adaptor sequences were used, A (5'-CGTATCGCCTCCCTCGCGCCA) and B (5'CTATGCGCCTTGCCAGCCCGC). Combinations of these adaptors with forward and reverse primers allowed the usage of a complete Roche amplification kit (Roche Diagnostics Nederland BV, Almere, The Netherlands) for unidirectional sequencing. The PCR amplicons were then checked by electrophoresis on 2% agarose gel and purified using the QIAquick PCR Purification Kit (Qiagen). DNA concentrations were determined using the Quant-iT™ PicoGreen^®^ dsDNA Assay Kit (Invitrogen, Gent, Belgium) and the amplicons were pooled together at an equal molar ratio. Thus, all amplicons from each primer set ended up in one multiplexed sample. The samples were pyrosequenced using a Roche 454 Life Sciences Genome Sequencer FLX machine at the VIB MicroArray Facility, KU Leuven. The GS FLX Titanium SV emPCR kit (Lib-A) (Roche Diagnostics Nederland BV, Almere, The Netherlands) was used for titrations, and the GS FLX Titanium MV emPCR kit (Lib-A) (Roche Diagnostics Nederland BV, Almere, The Netherlands) was used for amplification of DNA libraries. For pyrosequencing, the GS FLX Titanium Sequencing kit was used (Roche Diagnostics Nederland BV, Almere, The Netherlands).

### Calprotectin elisa assay

Cecal content of the mice was collected and kept at -80°C until used for this assay. Calprotectin elisa was performed using S100A8/S100A9 Elisa kit (ref K6936) from Immunodiagnostik (Immunodiagnostik, Bensheim, Germany) following the protocol suggested by the producer. The concentration of calprotectin was calculated from measured OD 450 nm values by the Gene5 program (Biotek, Winooski, VT, USA).

### Sequence analysis

Sequences were analyzed with the QIIME pipeline, version 1.4 [80]. After multiplexed sequencing of the 16S PCR products, sequences were assigned to samples based on their Molecular Identifier (MID) tag, allowing for one base error. Only 454 reads with a length > 200 bp and < 1,000 bp, an average quality score above 25, fewer than two ambiguous bases, and fewer than two primer mismatches were retained for further analysis. To remove sequencing errors, chimeric reads were identified and removed using ChimeraSlayer [56] with default settings. Chimera-cleaned reads were denoised using the QIIME integrated Denoiser and OTUs were subsequently clustered from denoised reads at a 97% identity threshold using uclust [81] with QIIME default settings. We retained 297,597 high quality reads for further analysis, with an average of 4,960 reads per sample, which were clustered into 593 OTUs. For each OTU, the most abundant sequence was selected as the representative read and classified using RDP classifier [82], only accepting annotations with at least 80% confidence. This way we could assign 99.5%, 98.8%, 97.5%, 93.9% and 37.7% of reads to phylum, order, class, family and genus levels, respectively. From OTU abundance and their respective taxonomic classifications, feature abundance matrices were calculated at different taxonomic levels, representing OTU and taxa abundance per sample. OTU counts per sample, OTU taxonomical assignments and metadata are available in Additional file 21.

### Statistical analyses

To compare the different sequence samples selected by the QIIME pipeline, sample counts were rarefied to 2,258 reads per sample for the initial two-cluster analysis and 3,700 for all other analysis steps. The rarefaction depth was chosen based on the 90% of the lowest sequencing depth over all included samples. For visualization of taxa abundances, taxa abundance was converted to a log10 scale by adding 1 to each taxa prior to transformation, avoiding infinite values for absent taxa. Statistical analysis was conducted on the rarefied feature abundance matrices using R 2.12.2.

For the initial sample stratifications we used Partioning around Medoids (pam) [58] to cluster samples based on four distance metrics: Jensen-Shannon [83], Bray-Curtis [84], Euclidean distance and weighted Unifrac [85]. Additionally, several other clustering algorithms were used to test for stable clustering, including k-means clustering (as implemented in the R package 'flexclust'), average, single, ward and complete hierarchical clustering (via the function 'hclust' in R). The distances were calculated from genus level normalized abundances, with the exception of Unifrac distances, which were calculated from OTU level by the Qiime pipeline. Optimal cluster number was calculated using either the Calinski-Harabasz pseudo F-statistic (using medoids as centers), Silhouette internal cluster optimality criterion, Baker and Hubert Gamma or the Davies-Bouldin's index, as implemented in the R package clusterSim. The density of samples along the NMDS axis was calculated using a Gaussian Kernel from the R 'density' function with default parameters. To test the stability of the clustering further, we used a resampled clustering of samples, leaving 10% of samples randomly out of clustering during each of the 500 repetitions. A second bootstrap test was used to randomly reassign the taxonomy of 10% of the OTUs and recalculate the genus abundance matrix from this set, which was also repeated 500 times. Samples that were in 97.5% of cases associated with the same cluster were considered to be stable. All ordinations (NMDS, dbRDA) and subsequent statistical analysis were calculated using the R-package vegan with Bray-Curtis distance on the rarefied and log-transformed taxa abundance and visualized with custom R scripts. Community differences were calculated using a permutation test on the respective NMDS reduced feature space, as implemented in vegan. Furthermore, we calculated intergroup differences for the microbiota using PERMANOVA [86] as implemented in vegan. This test compares the intragroup distances to the intergroup distances in a permutation scheme and thus calculates a *P*-value. For all PERMANOVA tests we used 5,000,000 randomizations. PERMANOVA *post hoc P*-values were corrected for multiple testing using the Benjamini-Hochberg false discovery rate (q-value) [87]. The variation explained by the genotype and cage effect factors was calculated using variation partitioning analysis [88] as implemented in the vegan R package, but modified to our specific setup (the original code does not support calculation of an unadjusted coefficient of determination (R^2^) for factors, which would in our case lead to each individual cage and genotype being treated as a separate regression to be adjusted for; this was solved by using unadjusted R squared values in agreement with the original developer of this package (Pierre Legendre, personal communication; code available upon request)). To calculate the variation explained by one group (that is, *Helicobacter*) within our dataset, we calculated variation explained on the complete community matrix and compared this to a matrix from which all *Helicobacter *OTUs had been removed. The differences between these two variation-partitionings was taken as the variation explained by *Helicobacter*, in the context of, for example, the cage effect.

To test for intragroup dispersion, inter-sample distances were calculated as described above and tested for equal intragroup dispersions using betadisper [89] as implemented in vegan; the significance was calculated using anova. Univariate testing for differential abundances of each taxonomic unit between two or more groups was tested using a Kruskal-Wallis test (*P*-value), corrected for multiple testing using the Benjamini-Hochberg false discovery rate (q-value) [87]. Taxa with less than ten reads over all samples were excluded from this analysis to avoid artifacts. *Post hoc *statistical testing for significant differences between all combinations of two groups was conducted only for taxa with a significance of *P *< 0.2. Wilcoxon rank-sum tests were calculated for all possible group combinations and corrected for multiple testing using Benjamini-Hochberg false discovery rate (q-value). Calprotectin correlations to taxa were tested using a spearman correlation test; *P*-values were corrected using Benjamini-Hochberg false discovery rate. For testing the influence of age, a blocked Spearman test as implemented in COIN [90] was used, where genotype was used as blocking factor. To delineate enterotype influence from cage/genotype effect, we used a blocked independence test as implemented in COIN [90].

Taxonomic richness was calculated by rarefying the respective non-normalized feature abundance matrices until 3,800 (90% of minimum read number) or in the case of the enterotype calculations rarefactions to 2,300 (several samples in the minor enterotype were below 3,800 reads) reads per sample. The number of different taxa was calculated for each rarefied sample. This was repeated five times per sample, and the average is the reported richness. Analogous to this, Chao1 [91] richness estimates and Shannon diversity [92] estimates were calculated from the rarefied OTU matrix. We tested for significant differences in observed richness, richness estimates or Shannon diversity using a Kruskal-Wallis test.

SNP genomic distances between mouse strains were obtained from [62]. The Bray-Curits microbiome distance between the strains for which genetic distances were available was calculated from the rarefied and transformed abundance matrix. Between strain distances were calculated from the median distance between all samples from the respective strains. The microbiome and genomic distance matrix were tested for correlation using Mantel's test [93]. Subsequently, a separate NMDS was calculated for each genomic and metagenomic distance, and a Procrustes transformation was used to visualize the similarities between these two ordinations.

### Data accession

Sequences have been deposited in the NCBI Short Read Archive [SRA054360].

## Abbreviations

bp: base pair; CH: Calinski-Harabasz; dbRDA: distance-based redundancy analysis; ET: enterotype; NMDS: nonmetric multidimensional scaling; NOD: non-obese diabetic; OTU: operational taxonomic unit; PCR: polymerase chain reaction; SNP: single-nucleotide polymorphism.

## Competing interests

The spouse of AL is an employee of UCB.

## Authors' contributions

AN and BB performed the experiments. FH, AN and RG analyzed the data. FH, BB, PV, AL and JR designed and conceived the experiments. AN, FH, AL and JR wrote the paper. All authors read and approved the final manuscript.

## Supplementary Material

Additional file 1 **Figure S1 - overview of gut microbiome composition of investigated samples at the phylum level**. Mouse strains are abreviated by the first letters and correspond in color to Figure 1a: N, NOD; F, FVB; BA, Balbc; S, Swiss; B, B6. Click here for file

Additional file 2 **Figure S2 - density plotting of samples on NMDS revealed two enterotypes at the phylum level**. The same result, that is, two optimal clusters, was observed when using three different distance matrices: **(a) **genus level Bray-Curtis, **(b) **genus level Jensen-Shannon and **(c) **OTU level weighted Unifrac. Click here for file

Additional file 3 **Table S1 - distribution of enterotypes among genotypes and cages**. Distribution of enterotypes among **(a) **genotypes and **(b) **cages. Enterotype 1 and 2 are labeled as ET1 and ET2, respectively. Click here for file

Additional file 4 **Table S2 - optimal clustering numbers of the total dataset**. **(a) **The optimal number of clusters obtained by Silhouette index/Calinski-Harabasz (CH) score. **(b) **The actual observed CH score. **(c) **The observed maximum Silhouette index. This is repeated at five taxonomic levels using four different distance methods. Note that Unifrac distance can only be measured at the OTU level. Click here for file

Additional file 5 **Table S3 - comparison of optimal cluster number under differing clustering methods as well as optimal cluster number scores**. All data are calculated at the genus level, using Jensen-Shannon distance. Abbrevations: CH, Calinski-Harabasz pseudo F-statistic; SIL, Silhouette internal cluster optimality criterion; BHG, Baker and Hubert Gamma; DB, Davies-Bouldin's index. Click here for file

Additional file 6 **Table S4 - 10% of the taxonomy was either resampled or the samples were jackniffed to 54 samples**. This was repeated 500 times under 5 clustering conditions using pam clustering and the taxonomic level as indicated. The optimal cluster number in these 500 resamplings is shown in the tables. Abbrevations: CH, Calinski-Harabasz pseudo F-statistic; SIL, Silhouette internal cluster. Click here for file

Additional file 7 **Table S5 - taxa that showed significant differences between enterotype 1 (ET1) and enterotype 2 (ET2)**. Cut-off values (*P *< 0.05 and q < 0.1) were applied. The OTUs were identified at the genus level if applicable. In case no genus or family could be identified, we took the lowest identified taxonomy. Click here for file

Additional file 8 **Table S6 - univariate test showing correlations between amount of calprotectin in cecal matter and gut bacteria**. Marked groups are those negatively linked to calprotectin amount (Rho < 0). Cut-off values are *P *< 0.05 and q < 0.1. Click here for file

Additional file 9 **Table S7 - *P*-values of genetic and cage effect calculated from NMDS analysis at all taxonomic levels**. The randomized test was limited to 10^4 ^permutations. Click here for file

Additional file 10 **Figure S3 - visualization of genetic and cage effects using distance-based redundancy analysis**. Genetic as well as cage effects show a strong correlation to the mice microbiome, as visualized in the dbRDA at the **(a) **phylum and **(b) **genus levels. Samples are colored by genotype; cages are visualized by connecting lines between samples. Click here for file

Additional file 11 **Table S8 - variation partitioning taking into account genotype, cage, and enterotype as well as shared information between these and unexplained variation**. Percentage of variation in microbiota composition explained by solely genotype, cage and enterotype or by shared effects of those variables. Click here for file

Additional file 12 **Figure S4 - intra-strain dispersion of investigated mouse genotypes**. Intra-strain dispersion was not significantly different between investigated genotypes, as shown here for genus level. Click here for file

Additional file 13 **Table S9 - PERMANOVA *post hoc *testing for significant differences of gut microbiota compositions between the five strains used**. PERMANOVA *post hoc *testing for significant differences of gut microbiota compositions between the five strains used. The marked values are significant (*P *< 0.05, q < 0.1). Click here for file

Additional file 14 **Figure S5 - richness estimates at the OTU level over study factors**. OTU richness estimated with a Chao1 estimator. **(a) **For genotypes significant differences in richness were observed. **(b) **Cage effect did not show any significant differences. Click here for file

Additional file 15 **Table S10 - list of taxa showing significant differences between genotypes**. List of taxa showing significant differences between genotypes (stratified for ET1). Cut-off values of *P *< 0.05 and q < 0.1 were applied. The direction column sorts genotypes by their median abundance, from largest to smallest. A *post hoc *test was applied to direct neighbors in this list, where ' > ' is q-value of the test < 0.1, ' > > ' is q < 0.05 and ' > > > ' is q < 0.01. Click here for file

Additional file 16 **Table S11 - average abundance of bacterial groups showing significant differences between mouse genotypes**. Values in brackets are standard deviations within the corresponding groups. Click here for file

Additional file 17 **Table S12 - bacterial groups showing significant differences between cages**. Summary of bacterial groups showing significant differences between cages (*P *< 0.05 and q < 0.1). Male mice were excluded from this test. The direction column sorts genotypes by their median abundance from largest to smallest. A *post hoc *test was applied to direct neighbors in this list, where '=' is q-value > 0.1. Click here for file

Additional file 18 **Table S13 - PERMANOVA tests for community differences between genotypes and cages after removal of all *Helicobacter *OTUs**. Click here for file

Additional file 19 **Table S14 - blocked Kruskal-Wallis test on all samples**. **(a) **Blocked Kruskal-Wallis test on all (60) samples with enterotype as confounding factor yielded similar results, that is, bacterial groups showing significant difference between a) genotypes and b) cages as if enterotype had been pre-stratified. Click here for file

Additional file 20 **Figure S6 - schematic presentation of primer design used in the amplification of the V3-V5 region of 16SrDNA in this study**. Click here for file

Additional file 21 **Table S15 - metadata of all mice used in the study, the OTU abundance of all samples and the OTU taxonomical assignments**. Click here for file
