# Supplementary figures and images for: Inflammation-associated enterotypes, host genotype, cage and inter-individual effects drive gut microbiota variation in common laboratory mice
Source: Genome Biol. 2013 Jan 24;14(1):R4. doi: 10.1186/gb-2013-14-1-r4 (PMC4053703; doi:10.1186/gb-2013-14-1-r4)

# Supplementary Figure S1

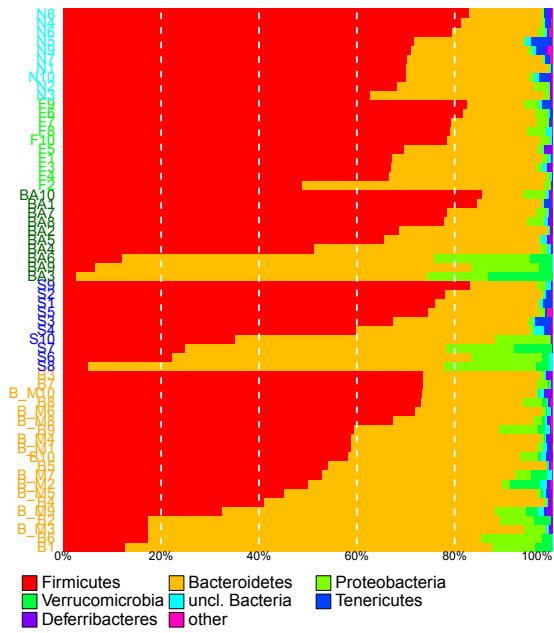

Supplement: Additional file 1 — Figure S1 - overview of gut microbiome composition of investigated samples at the phylum level. Mouse strains are abreviated by the first letters and correspond in color to Figure 1a: N, NOD; F, FVB; BA, Balbc; S, Swiss; B, B6. [file gb-2013-14-1-r4-S1.PDF]

Supplementary Figure S2

A Bray-Curtis

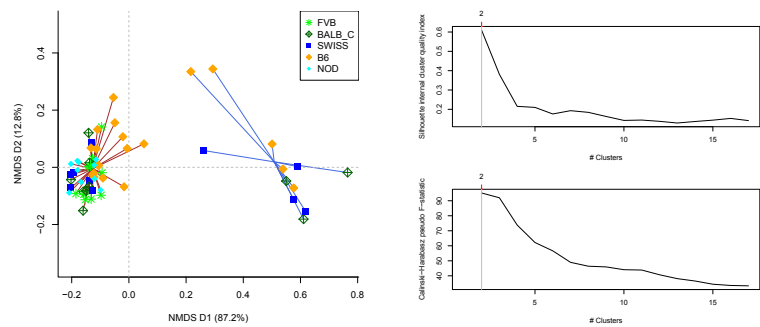

B Jensen-Shannon

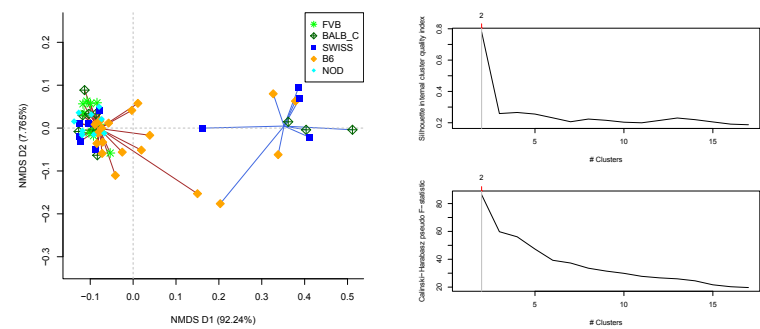

C weighted Unifrac

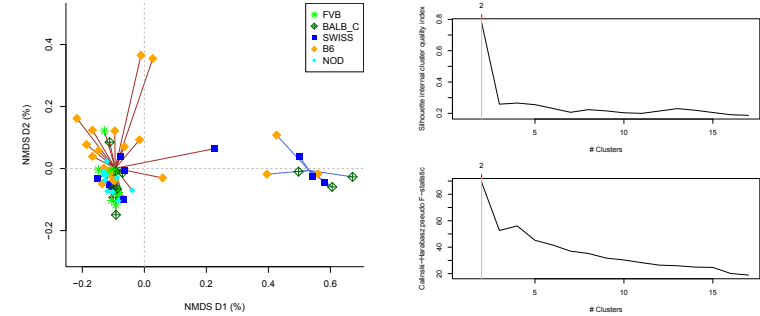

Supplement: Additional file 2 — Figure S2 - density plotting of samples on NMDS revealed two enterotypes at the phylum level. The same result, that is, two optimal clusters, was observed when using three different distance matrices: (a) genus level Bray-Curtis, (b) genus level Jensen-Shannon and (c) OTU level weighted Unifrac. [file gb-2013-14-1-r4-S2.PDF]

Supplementary Figure S3

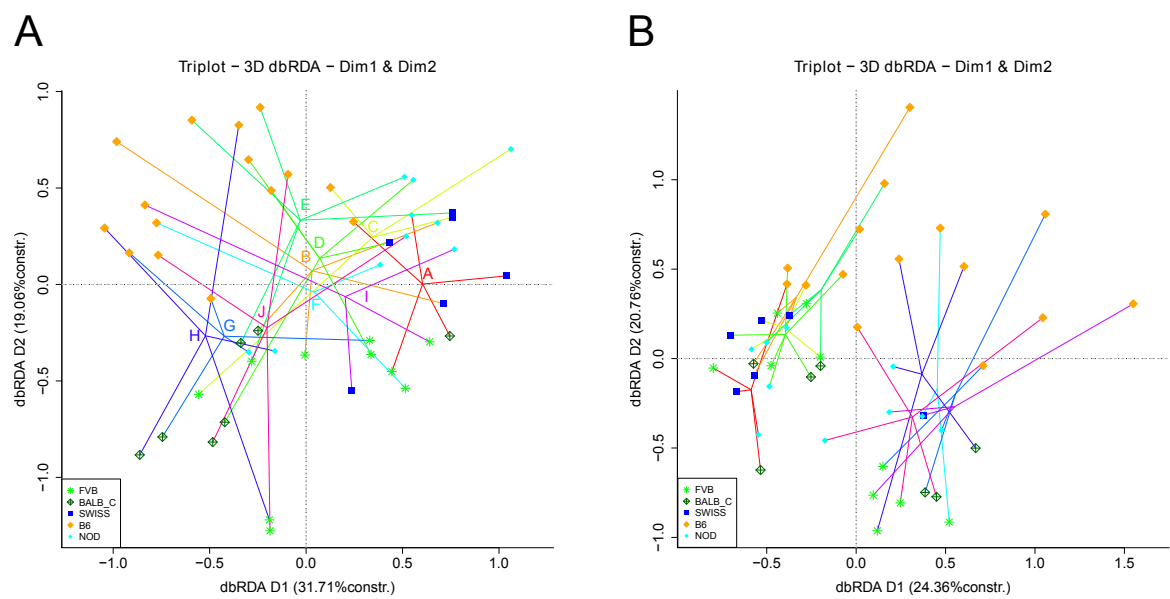

Supplement: Additional file 10 — Figure S3 - visualization of genetic and cage effects using distance-based redundancy analysis. Genetic as well as cage effects show a strong correlation to the mice microbiome, as visualized in the dbRDA at the (a) phylum and (b) genus levels. Samples are colored by genotype; cages are visualized by connecting lines between samples. [file gb-2013-14-1-r4-S10.PDF]

Supplementary Figure S4

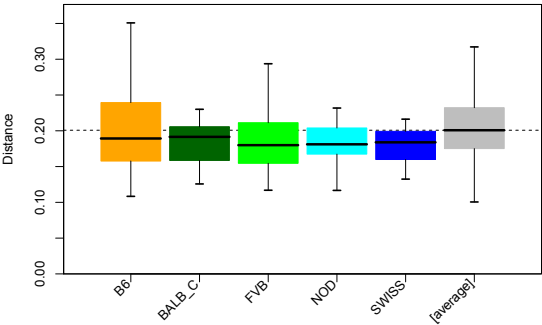

Supplement: Additional file 12 — Figure S4 - intra-strain dispersion of investigated mouse genotypes. Intra-strain dispersion was not significantly different between investigated genotypes, as shown here for genus level. [file gb-2013-14-1-r4-S12.PDF]

Supplementary Figure S5

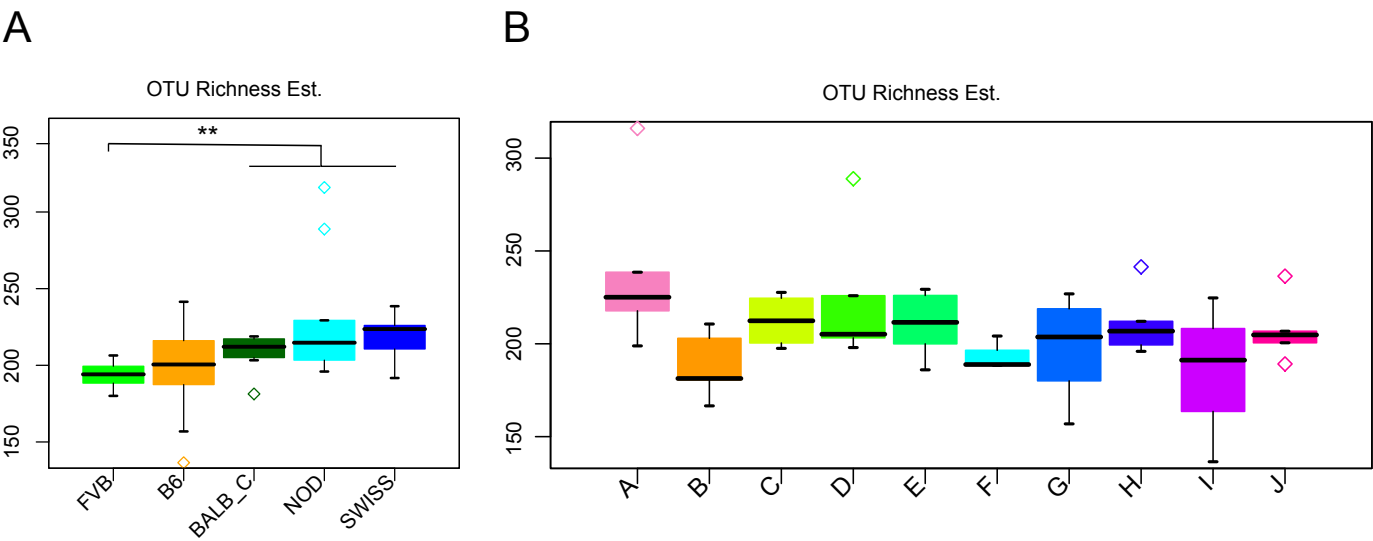

Supplement: Additional file 14 — Figure S5 - richness estimates at the OTU level over study factors. OTU richness estimated with a Chao1 estimator. (a) For genotypes significant differences in richness were observed. (b) Cage effect did not show any significant differences. [file gb-2013-14-1-r4-S14.PDF]
