# Supplementary material for: Inflammation-associated enterotypes, host genotype, cage and inter-individual effects drive gut microbiota variation in common laboratory mice
Source: Genome Biol. 2013 Jan 24;14(1):R4. doi: 10.1186/gb-2013-14-1-r4 (PMC4053703; doi:10.1186/gb-2013-14-1-r4)
Supplement: Additional file 20 — Figure S6 - schematic presentation of primer design used in the amplification of the V3-V5 region of 16SrDNA in this study. [file gb-2013-14-1-r4-S20.PDF]

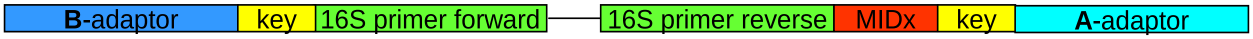


| primername | A-adaptor reverse | key | MID | 926 primer reverse |
|---|---|---|---|---|
| Arev_MID-1 | 5' CGTATCGCCTCCCTCGCGCCA | TCAG | ACGAGTGCGT | CCGTCAATTCMTTTRAGT |

| primername | B-adaptor forward | key | 357 primer forward |
|---|---|---|---|
| Bfor | 5' CTATGCGCCTTGCCAGCCCGC | TCAG | CCTACGGGAGGCAGCAG |

Additional file 14 Figure S6
